# Subtype-specific CpG island shore methylation and mutation patterns in 30 breast cancer cell lines

**DOI:** 10.1186/s12918-016-0356-2

**Published:** 2016-12-23

**Authors:** Heejoon Chae, Sangseon Lee, Kenneth P. Nephew, Sun Kim

**Affiliations:** 1 School of Informatics and Computing, Indiana University Bloomington, IN 47405, USA, Waterloo Road, Bloomington, IN, 47405 USA; 2 Department of Computer Science and Engineering, Seoul National University, Seoul, Republic of Korea; 3 Indiana University School of Medicine, Department of Cellular and Integrative Physiology, Medical Sciences Program, Bloomington, USA; 4 Interdisciplinary Program in Bioinformatics, Seoul National University, Seoul, Republic of Korea; 5 Bioinformatics Institute, Seoul National University, Seoul, Republic of Korea

**Keywords:** Breast cancer, Subtype, DNA methylation, CpGI shore, Mutation

## Abstract

**Background:**

Aberrant epigenetic modifications, including DNA methylation, are key regulators of gene activity in tumorigenesis. Breast cancer is a heterogeneous disease, and large-scale analyses indicate that tumor from normal and benign tissues, as well as molecular subtypes of breast cancer, can be distinguished based on their distinct genomic, transcriptomic, and epigenomic profiles. In this study, we used affinity-based methylation sequencing data in 30 breast cancer cell lines representing functionally distinct cancer subtypes to investigate methylation and mutation patterns at the whole genome level.

**Results:**

Our analysis revealed significant differences in CpG island (CpGI) shore methylation and mutation patterns among breast cancer subtypes. In particular, the basal-like B type, a highly aggressive form of the disease, displayed distinct CpGI shore hypomethylation patterns that were significantly associated with downstream gene regulation. We determined that mutation rates at CpG sites were highly correlated with DNA methylation status and observed distinct mutation rates among the breast cancer subtypes. These findings were validated by using targeted bisulfite sequencing of differentially expressed genes (*n*=85) among the cell lines.

**Conclusions:**

Our results suggest that alterations in DNA methylation play critical roles in gene regulatory process as well as cytosine substitution rates at CpG sites in molecular subtypes of breast cancer.

**Electronic supplementary material:**

The online version of this article (doi:10.1186/s12918-016-0356-2) contains supplementary material, which is available to authorized users.

## Background

Breast cancer is a diverse disease consisting of multiple different molecular subtypes, such as luminal A, luminal B, triple negative/basal-like, HER2-positive, and normal breast [1]. As these subtypes are associated with differences in clinical outcomes [2], more completely describing the precise molecular nature of breast cancer may eventually allow for “personalized” clinical decisions, translating molecular information into better treatments for patients with breast cancer [3]. In this regard, gene expression patterns have been widely used not only to identify breast cancer subtypes and but also to develop clinically useful gene signatures. Microarray-based transcriptional profiling identified 50 genes used for a classifier called PAM50 (Prosigna) [4]. The 21-gene assay Oncotype DX is predictive of breast cancer recurrence and the use of this 21-gene assay has a significant impact on treatment decisions [5].

Beyond gene expression profiling, epigenetic modifications, reversible, heritable and includes changes in DNA methylation, modification of histones and altered microRNA expression levels, have received recent attention in breast cancer subtypes [6, 7]. DNA methylation patterns in particular have been used to distinguish breast cancer phenotypes [8–13], and differentially methylated regions (DMRs) as prognostic breast cancer biomarkers (patient survival analysis) have been described [14, 15]. Furthermore, based on the association of DNA methylation with altered gene expression, a number of “integrated” DNA methylation/gene expression analyses have been performed, including those by Feinberg and co-workers (2009) [16] demonstrating the importance of methylation in areas surrounding CpG island (CpGI) shores, Brenet et al. [17] reporting the importance of 1st exon methylation, Sproul et al. (2011) [18] on the role of aberrant CpGI methylation and transcriptional repression in breast cancer lineages, our recent reports integrating DNA methylation and gene expression in breast cancer [19, 20]. However, a comparative analysis of DNA methylation at CpGI, CpGI promoters, and CpGI shores regions, more specifically at transcription binding site (TFBS) associated overlapped regions and their impact on gene expression in breast cancer molecular subtypes on a genome-wide level has not been reported.

Gene mutations are key events in cancer development, and recent cancer genome projects have yielded extensive comparisons of the mutational landscape in breast cancer subtypes [11] and mutations associated with clinical outcomes [21, 22]. In addition, complex relationships between mutation prevalence and transcription [23], as well as an association between DNA methylation and gene mutations [24, 25] have been reported. Recently, in [19], we reported that genome-wide methylation profiles were distinct among breast cancer subtypes and there were methylated sites in the promotor regions of genes that were down-regulated in a cancer subtype specifically way, suggesting that the methylated sites interfered interactions between transcription factors and the promotor genomic regions. However, this study did not report signatures of methylation in specific genomic regions for breast cancer subtypes and did not investigate relationship between DNA methylation and gene mutation rates among breast cancer molecular subtypes. By integrating methylation and mutation patterns, we demonstrated that:

1. Differential CpGI shore methylation patterns were characteristic of the basal B subtype. Furthermore, within CpGI shores, methylation at TFBSs and overlapping promoter CpGI regions was associated with differential gene regulation in basal B compared to other breast cancer subtypes.
2. Basal A breast cancer cells showed higher mutation rates at CpG sites with low or intermediate methylation, whereas mutation rates were higher at hypermethylated CpG sites in the basal B subtype.

## Motivation

This work was motivated by our previous works in modeling DNA methylation susceptibility [26–28] and conservation of CpG island sequences [29]. We and many scientists believe that DNA methylation is not random and probably there is an instructive mechanisms embedded in the genomic sequences [30]. Thus our motivation is to investigate where there is any notable correlation between mutations (cancer-subtype specific genomic sequences) and cancer subtype specific methylation patterns. In fact, there is recent article that suggests associations between mutations and epigenetic changes [31]. Thus our goal in this study is to look for any association between genome sequence differences and methylation patterns.

## Methods

### breast cancer cell line and subtype difference estimation

Genome wide DNA methylation status was measured in our previous work [19] by MBDCap sequencing from 30 breast cancer cell lines representing three different molecular subtypes; basal A, basal B and luminal obtained from (see Additional file 1: Supplementary Table S1 for more information on cell lines). MBDCap-seq utilizes affinity between MBD protein and methylated DNA sequence and allows cost-efficient measurement of genome wide DNA methylation status. Initial quality trimming is performed by Trim Galore [32] to remove bad sequence quality reads, and remained reads were aligned to reference genome (build hg19) by using Bowtie2 [33] with seed length 22 and allowing zero mismatch in it. Multiple and duplicated reads are then filtered out to mediate the possible PCR amplication bias. Aligned reads were counted through genome-wide scanning with 100bp length window by using MEDIPS, a R package providing fixed-length bin methylation estimation from affinity based sequencing data in the form of relative methylation score (RMS) [34]. The RMS value of each 100bp bin was then compared across the tumor subtypes to extract DMRs and their significance were tested by t-test with adjusted *P*-value (Bonferroni) < 0.05.

Affymetrix microarray based gene expression data was downloaded from [35] and expression level is measured by R Limma package [36] in Bioconductor. Background correction and normalization is performed on signal intensity to measure expression, and pair-wise and three classes subtype gene expression comparison was performed to extract differentially expressed genes (DEG)s. For the pair-wise gene expression comparison, linear model based Limma was used, and for three class comparison, mutual information based DEGPack [37] was used.

Normal breast control data were obtained from TCGA data portal (measured by whole genome bisulfite sequencing (WGBS); id: TCGA-A7-A0CE-11A-21D-A148-05) and from genome wide methylome study [14] (measured by MBDcap sequencing). Initial quality trimming and aligning were performed on both data set, and genome wide methylation status of TCGA WGBS data and MBDCap sequencing data was measured by methylKit [38] and MEDIPS [34] respectively.

### Targeted bisulfite-treatment sequencing

Our previous work [19] used MBDCap sequencing data without bisulfite treatment. Thus we performed targeted bisulfite sequencing on 85 gene regions. Among 30 breast cancer cell lines, six samples (two lines representing each subtypes; see Additional file 1: Supplementary Table S1) were selected for targeted bisulfite treatment sequencing validation. Pre-library preparation utilized 3 *μ*g DNA and all libraries passed a minimum fragment size of 200 to 250 bp and ≥147 ng/ *μ*l quality control. Hybridization was performed using SureSelect^*TX*^ Methyl-Seq Kit followed by post library generation with targeted genomic region information. Final library concentration was 250 >pM. Based on the captured library, bisulfite conversion was performed to distinguish methylated and unmethylated DNA regions.

Sequencing was performed on 85 distinct DEG regions with additional 10 Kbp upstream of transcription start site (TSS) using Illumina HiSeq2500. A total of 300 million reads were aligned to reference genome (build hg19) with bisulfite conversion by using Bismark [39], and each CpG site methylation was measured by using methylKit [38].

### Correlation between targeted bisulfite-treatment sequencing and MBDcap sequencing

Affinity based MBDcap sequencing captures methylated reads and number of mapped reads at certain range represents the methylation status on that. On the other hand, bisulfite treatment converts only un-methylated cytosine to uracil and given that information it provides methylation level in single base pair resolution. In order to estimate the correlation between methylation levels measured by BS seq and MBDcap seq, genome-wide single base pair read coverage was measured from MBDcap seq data. Then, CpG site read coverage was extracted and intersected with targeted bisulfite treated regions to filter out result from other regions. Lastly, 2 kb bin methylation level were computed on both methods, and Pearson’s correlation was estimated between them.

### Experimentally validated transcription factor binding site and their methylation status

In search of the specific transcription factor binding sites (TFBSs) located in CpGI shores and the overlapping promoter region, we utilized match algorithm from TRANSFAC [40]. Promoter sequences were extracted from 2 Kb upstream of the TSS in each DEGs, and TF motif weighted matrices were used to scan the TFBSs on the sequence regions. Once TFBSs were predicted, we computed the TFBS specific methylation level by averaging methylation levels in all 100 bp bins overlapping the TFBS. Finally, we adopted experimentally validated ChiP-seq databases (HTRIdb [41], and ChEA [42]) to verify TF binding on predicted TFBSs. In order to investigate potential downstream effect caused by methylation difference on TFBS, differential methylation across tumor subtypes was measured on TFBSs by Kruskal Wallis test (FDR < 0.1) and correlation with downstream gene was estimated (Spearman’s rho < -0.5). To remove effect of TFs on gene regulation, we considered only TFs with similar gene expression levels, allowing us to focus on the role of DNA methylation on downstream genes.

### Mutation rate and subtype specific mutation

MBDcap-seq is a DNA sequencing technology capturing methylated regions by utilizing affinity between MBD protein and methylated DNA sequence. To investigate the relationship between methyl-CpG mutation and their methylation level, genome wide point mutation discovery (matches short reads to the hg19 build) was performed on MBDcap-seq data by using the mpileup algorithm in the Samtools suite (version 0.1.19) [43]. Minimum base quality for a base to be considered was set to 13, and maximum reads per sample was set to 250. By incorporating sequence and quality information and mismatch sharing rates across the samples, every read having mismatches with the reference genome was statistically tested to determine whether or not the observation was due to sequencing error. In order to reduce false mutation detection caused by misaligments and indel, base alignment quality (BAQ), Phred-scaled probability of a read based being misaligned, is applied to each base [44]. In addition, anomalous read pairs in variant calling were skipped. Finally, mutation rates within a certain methylation range across the tumor subtypes were computed. We defined mutation rate as the ratio of number of cytosine substitution occurrence over the number of all CpG sites. In order to estimate statistical significant of computed mutation rate within certain methylation range, the mutation rate information is pooled into subtypes and tested by ANOVA with Bonferroni correction. In addition to detecting variants from all samples, subtype specific mutations were also measured. Each detected mutation was checked as to whether the observation was from all samples or only certain tumor subtype samples. We defined a subtype specific mutations as those that occurred in at least 30% of a particular subtype but in less than 10% of the other two subtypes.

**Fig. 1:**
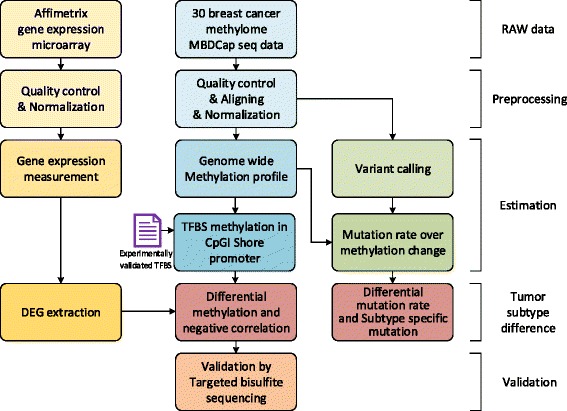
Workflow for the methylation and mutation analysis of 30 breast cancer cell lines. A total of 30 breast cancer cell lines representing molecular subtypes of the disease were examined in this study. Analysis starts with quality control and normalization on both MBDcap sequencing data and Affimatrix gene expression data, and methylation and expression level were measured. During integrated analysis, subtype comparison was performed to estimate differentially expressed genes (DEG)s and differentially methylated regions (DMR)s. Experimentally validated transcription factor binding site (TFBS) information is used to estimate TFBS specific methylation level in promoter and CpGI shore overlapped region, and correlation was measured with downstream gene expression. By utilizing mutation information estimated from MBDcap sequencing, subtype specific mutation rate over methylation level was measured. Finally, single base pair resolution bisulfite treatment sequencing was performed to validated the methylation status measured by MBDcap sequencing

Whole schematic analysis workflow is illustrated in Fig. 1.

## Results

### Genome wide methylation profile and differentially methylated regions

**Fig. 2:**
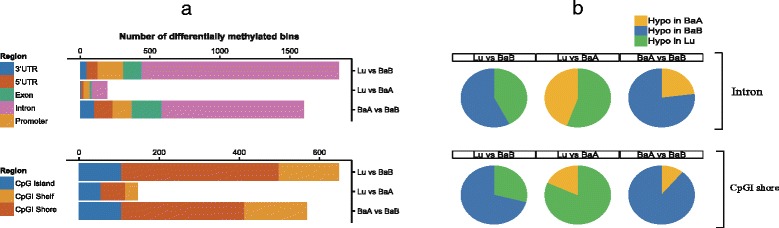
**a** Bar plots demonstrate the number of differentially methylated bins for each pair-wise tumor type comparisons. Significance of each bin methylation on each genomic region between two subtypes are tested by t-test and adjusted with Bonferroni correction (P.adj-value < 0.05). **b** Ratio of hypo methylation in intron and CpGI shore regions. Each color represents hypo methylation ratio of certain tumor subtype among differentially methylated bins. Hypo methylation ratio of other regions are in Supplementary Figure S1 (see Additional file 1)

Genome wide methylation landscape was determined in 30 breast cancer cell lines MBDCap-seq. Methylation profiling using more than 30 million reads covered 23,149,286 CpG sites, 25,974 CpG islands, 54,543 CpGI shores, and 38,208 promoter regions (82, 91, 95, and 99% of the total in the human genome, respectively), and for overlapped regions, 10,910 promoter-CpGI and 16,227 promoter-CpGI shores (90 and 98% of total in human genome) were covered. A total 4,366 differentially methylated 100bp-bins corresponding to 2,055 differentially methylated regions (DMRs; MEDIPS package, adjusted *P*-value (Bonferroni) < 0.05) were determined (see Methods). 126 DMRs were identified in the luminal and basal A pair, 1,136 in the luminal and basal B pair, and 793 in the basal A and basal B pair. Statistics of differentially methylated bins were further grouped according to the genomic regions such as 3^′^UTR, 5^′^UTR, exon, intron, promoter, CpGI, CpGI shelf, and CpGI shore. Notably large number of differentially methylated bins were observed in intron and CpGI shore region from Lu-BaB pair and BaA-BaB pair (Fig. 2 a). Then, based on these comparison results, hypomethylation ratio of each subtype was further measured. In both intron and CpGI shore region, more than 75% (BaA-BaB pair) and 50% (Lu-BaB pair) of differentially methylated bins are hypomethylated in Basal B subtype (Fig. 2 b). Hypomethylation ratio of other regions are in Supplementary Figure S1 (see Additional file 1).

### Methylation status validation by targeted bisulfite sequencing

Affinity-based MBDCap-seq technology is a cost-efficient method to estimate genome-wide DNA methylation. However, it does not measure methylation level at the single nucleotide resolution, especially in high CpG density regions. In order to verify the methylation level estimated by MBDCap-seq method, we conducted targeted bisulfite-treated sequencing (BS-seq) on the genome regions around significantly differentially expressed genes (DEGs) (from 10Kbp upstream of the TSS to transcription end site (TES) including the corresponding promoter; see details in Methods). We compared methylation levels estimated from MBDcap-seq and BS-seq, and observed a strong average correlation (Pearson’s correlation coefficient 0.77) and up to 0.91 between two techniques (see Additional file 1: Figure S2), demonstrating that MBDCap-seq reliably measured genome wide methylation levels.

### Global CpGI shore hypomethylation specific to basal B tumor type

**Fig. 3:**
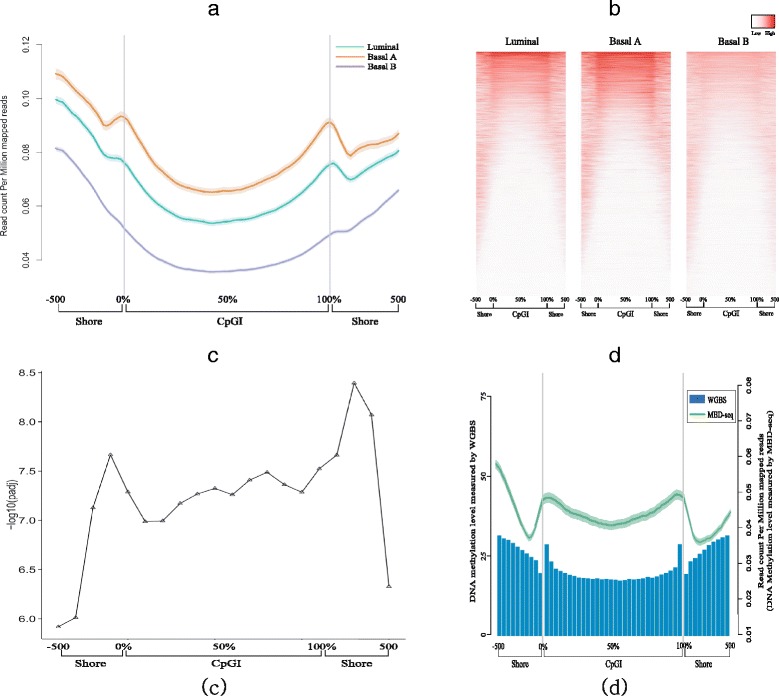
Genome wide profiling identifies differences methylation status among breast cancer subtypes. **a** Average genome wide methylation plot of CpG islands and flanking regions, i.e., CpGI shores. -500, 500 in *x-axis* represents ±500bp from each end of CpGI, and 0, 50, 100% represents relative range within CpGI. **b** Heatmap demonstrates average methylation levels of each CpG island and CpGI shore within tumor subtypes. **c** Statistical significance of differential methylation at CpG islands and CpG island shore regions tested by ANOVA and adjusted by Bonferroni correction. Y-axis represents -log10 based adjust *P*-value. **d** *Bar plot* and *line plot* represent average methylation level of adjacent area between CpGI and CpGI shore region from a normal-like TCGA breast cancer sample, a invasive ductal carcinoma with negative margins for malignancy, measured by WGBS and from a normal sample obtained from [14] study measured by MBDcap sequencing respectively. *Left* and *right* side of *y-axis* shows methylation level measured by WGBS and MBDcap sequencing respectively

Based on the genome-wide methylome analysis using affinity based MBDcap-seq data, we observed genome wide hypomethylation in the basal B subtype at various genomic regions. The average methylation levels in genebody, exon, as well as Dnase I hypersensitive sites were lowest in basal B (see Additional file 1: Figure S3). In addition, significant differential methylation patterns were observed in boundary areas between CpGI and CpGI shore. Notably, while methylation level peaks were observed in luminal and basal A, the steep peaks tapered into a gentle slope or nearly flattened out in basal B (Fig. 3 a). In addition, from heatmap for genome wide CpGI and their flanking area, highly methylated boundary region in luminal and basal A are observed, but not from basal B (Fig. 3 b). Significance of differential methylation among subtypes in entire CpGI and their flanking regions was tested by ANOVA and the *P*-value is adjusted by Bonferroni correction. From the result of statistical significance estimation, we identified that adjacent regions between CpGI and CpGI shore area have significantly low adjusted *P*-value compared to near regions (Fig. 3 c).

In order to further validate the observed methylation patterns, we utilized two normal data set; TCGA normal breast data measured by WGBS and normal data from genome wide methylome analysis study [14] measured by MBDcap sequencing. Genome wide methylation level were estimated through same analysis procedure (see “Methods”) and estimated average methylation in CpGI and CpGI shore regions. From methylation result based on both normal data, we observed same pattern and found steep peaks as well in adjacent region between CpGI and CpGI shore (Fig. 3 d).

To investigate whether the differences in methylation patterns in CpGI and adjacent region CpGI shore potentially involved in gene regulation, we focused on promoter CpGI shore with transcription factor binding site (TFBS). Estimated TFBS specific methylation status (see “Methods”) in the promoter CpGI shore was compared with downstream gene expression, and the TF binding to these TFBS regions was also measured to determine whether a TF influences gene regulation. That is, we investigated whether the differentially methylated TFBS in promoter CpGI shore regions among breast cancer subtypes potentially give influence to expression of downstream genes that TF regulate.

**Fig. 4:**
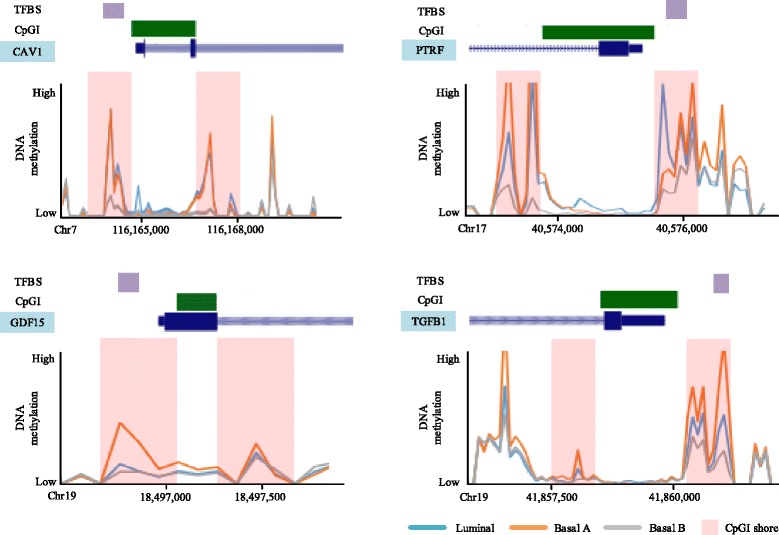
Differentially methylated and experimentally validated promotor TFBS in CpGI shore region having negative correlation with downstream gene expression. The differential methylation of the overlapped region among breast cancer cell subtypes was tested by Kruskal Wallis test with FDR < 0.1, and the methylation status was inversely correlated with downstream gene expression (Spearman rank correlation < -0.5). *X-axis* shows genomic location of each genes and *Y-axis* represents DNA methylation level measured by MBDcap sequencing

We identified 55 genes with differentially methylated promoter TFBS regions (Kruskal Wallis test, FDR < 0.1) and inversely correlated (Spearman’s rho < -0.5) gene expression (see Additional file 1: Table S2). Interestingly, 55% of these genes were hypomethylated in basal B, including CAV1 and PTRF (caveolae associated protein coding genes). Epigenetic modification of these caveolae related genes was recently reported to be associated with disease [45]. Furthermore, a significant influence of CpGI shore methylation on CAV1 in breast cancer was previously reported [20]. We confirmed this finding, detecting a significant DMR within the CpGI shore overlapping the CAV1 promoter (Fig. 4). We then further investigated the methylation status of TFBS located in the CAV1 promoter and the overlapping CpGI shore region. Interestingly, the experimentally validated TFBS regions showed significant differential methylation (Kruskal Wallis test, FDR < 0.005). In addition to CAV1, promoter and CpGI shore methylation with TFBS of PTRF, TGFB1, and GDF15 genes are depicted in Fig. 4. All these TFBS specific methylation within promoter and CpGI shore overlapped had inverse correlation with downstream gene expression that the TFs associated. Finally, CpGI shore methylation was validated (single base pair resolution) using targeted bisulfite sequencing (see Additional file 1: Figure S4).

### Correlation between mutation and methylation across molecular tumor subtypes

**Fig. 5:**
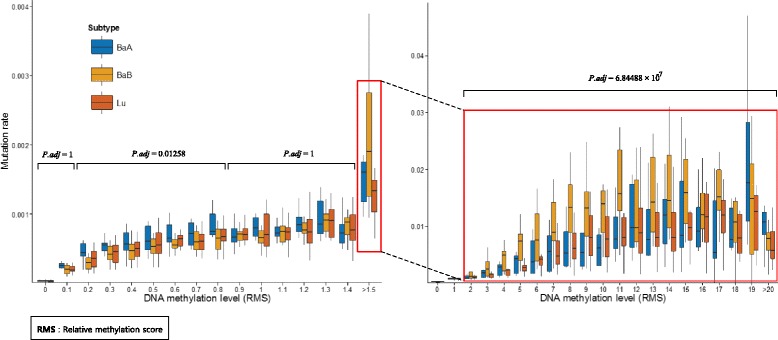
Significantly higher mutation rate in low and intermediately methylated CpG sites in basal A (ANOVA test, adjusted *P*-value (Bonferroni) < 0.05) whereas significantly higher mutation rate in hypermethylated CpG sites in basal B (ANOVA test, adjusted *P*-value (Bonferroni) < 0.05). *X-axis* represents each methylation level (RMS) value and *y-axis* represents ratio of mutational CpG site over all CpG site at certain methylation level. *Box plot* to the right: extension of *red box* area

In order to investigate the relationship between mutations and methylation variation, cytosine substitution rate on CpG site was computed across the tumor subtypes. By comparing the genome wide methylation profile and estimated mutation frequencies, the mutation rate gradually changed as the methylation level increased in all samples. We then compared mutation rates across the tumor subtypes and found that the mutation rate pattern over the methylation change was significantly different in different subtypes. At CpG sites displaying low and intermediate methylation, luminal and basal B had similar mutation rate but basal A showed a distinct and higher mutation rate (*P*-value = 1.1258 ×10^−2^ by ANOVA test with Bonferroni correction). Conversely, at highly methylated CpG sites, luminal and basal A had similar mutation rates but the mutation rate was significantly different (*P*-value = 6.84 ×10^−7^ by ANOVA test with Bonferroni correction) for the basal B subtype (Fig. 5).

**Fig. 6:**
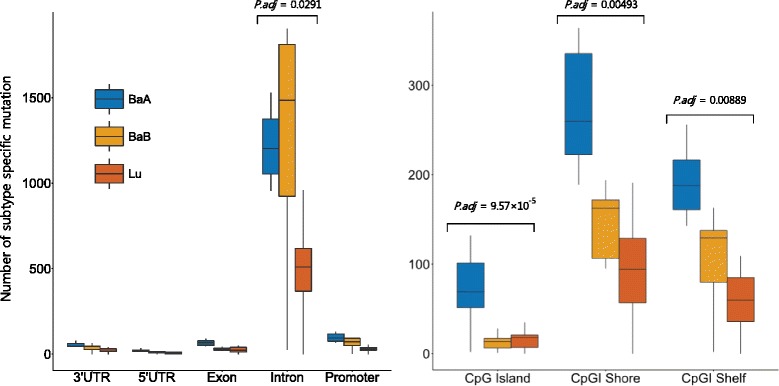
Subtype specific mutation occurrence associated with tumor subtypes across the genomic regions. Significance of difference among subtype specific mutation occurrent was tested by ANOVA with Bonferroni correction. (P.adj < 0.05). In intron region significanltly more subtype specific mutation is occurred in Basal B. On the other hand, In CpGI related regions, significantly more subtype mutation is observed in Basal A tumor subtypes. *X-axis* represents each genomic regions and *y-axis* shows number of subtype specific mutation occurred in those regions

To find possible biological explanation of the observed mutation rate difference across the tumor subtypes, we investigated whether there were any regional genomic effects. We first divided observed mutations by various regional groups based on their genomic position information. We then extracted subtype specific mutations, a mutation that occurs frequently in one subtype but rarely observed from others, by filtering out common mutation over all subtypes in each regional group (see Methods). Interestingly, in CpGI regions (known as “methyl protected” and thus hypomethylated regions) including CpGI shore and shelf, basal A specific mutations occurred the most frequently, and CpGI shore and shelf region showed significant differential subtype specific mutation occurrence (tested by ANOVA with adjusted *P*-value (Bonferroni) < 0.05). On the other hand, basal B specific mutations were significantly more frequent in intron regions (ANOVA, P.adj (Bonferroni) < 0.05) (Fig. 6). Our analysis suggests that mutation rate difference may result from regional subtype specific mutation occurrence and their methylation difference across the subtypes.

## Discussion

In this study, we report two novel findings associated with tumor subtype differences in terms of methylation and mutations. For the methylation pattern, we showed that CpGI shore methylation is a distinct signature for breast cancer subtypes and also that CpGI shore methylation is associated with subtype specific gene regulation. For the subtype specific methylation patterns, there are a number of studies. Previously, Holm et al., showed that unsupervised methylation pattern analysis could distinguish molecular subtypes [9]. Jadhav et al., reported differential methylation patterns in promoter CpGI, intragenic and intergenic CpGI as well as non-CpGI promoter regions compared to normal samples [46] and Kamalakaran et al., reported differential methylation pattern and association with clinical variable in luminal subtype [47]. More recently, Stefansson et al., tried to define additional epigenetic subtypes based on differential methylation patterns [13]. In agreement with previous studies, we observed significant differential methylation pattern on CpGI shore and promoter overlapping regions. Our further analysis on TFBS specific methylation revealed strong inverse correlation to downstream genes. We also detected more prevalent hypomethylated DMR bins in intron region for basal B subtype and this finding is in agreement with previously described genebody hypomethylation pattern studied by Yang et al. [48]. This genebody hypomethylation phenotype is also linked to hormone-receptor negative/basal-like breast cancers as described in Hon et al. [49].

In addition to genome wide differential methylation pattern, our integrated analysis identified genes having significant differential methylation on their TFBS located in promoter CpGI shore region, and having inverse correlation with their gene expression. CAV1 and PTRF are previously reported as cancer-associated caveolae genes [20]. GDF15 and TGFB1 genes are members of transforming growth factor beta family, and encode multifunctional proteins associated with proliferation, differentiation, adhesion, and migration. Therapeutically, these genes are related to response of breast cancer cells to radiation, specifically inhibiting radiation-induced cell death and related cytotoxic action [50] and a direct association between promoter methylation and expression of these genes are reported [51]. In addition, integrated analysis showed GSTP1 and PALLD genes having low level gene expression as well as significantly higher methylation level of these gene promoters in luminal phenotype compared to the other two subtypes. Hypermethylation of the GSTP1 promoter has also been previously reported as having association with prognostic values [52], and repression of PALLD gene has been shown to contribute to invasive motility [53] and cancer cell migration [54]. Including these genes, a large number of detected genes from our analysis have overlapping of promoter regions with DHS region as well as polycom-associated H3K27me3 marked region, suggesting a potential interplay with gene transcription and that differential methylation may play important roles across the subtypes.

Mutations play an important role in the development of cancer. Several studies investigated relationship between DNA methylation and mutation. Carina et al. reported a relationship between CpG cytosine mutation rates in intron regions in human genes and variation in methylation levels as well as a positive correlation with non-CpG divergences, and a negative correlation with GC content [55]. In another study focusing on exonic regions [24], methylation in first exon regions significantly correlated with C to T substitution rate in CpG sites. Based on genome wide mutation rate measurements, CpG sites with low-to-intermediate methylation level had higher CpG substitution rates compared to other methylated CpG sites [25]. Our genome wide mutation rate analysis shows notable differences in mutation rates across the tumor subtypes, which correlates with methylation status. In summary, our findings on mutation and methylation indicates a trend for higher mutation rates in basal A type at low to intermediate methylation level CpG sites whereas in the basal B phenotype, mutation rates are higher at highly methylated CpG sites.

## Conclusion

By utilizing methylome data and gene expression for 30 breast cancer cell lines, we report two novel findings. First, our genome wide integrated analysis shows significant difference in the CpGI shore methylation pattern among breast cancer molecular subtypes. Further investigation of these regions identified 55 genes with differentially methylated promoter regions overlapping CpGI shore regions with an inverse correlation of methylation level and transcriptional regulation of these 55 genes, but no apparent difference in expression of TFs that could potentially interact with their promoter CpGI regions. This consideration of TF and TFBS provides strong evidence for the suppressive role of DNA methylation on the downstream genes. Second, we found a genome-wide relationships between mutation rate and methylation level in the molecular subtypes. From the integrated analysis, we report that mutation rate gradually increases as methylation level increases. We further investigated this pattern in relation with the molecular subtypes and found higher mutation rates in basal A when the methylation level is low-to-intermediate, but basal B breast cancer cells have higher mutation rates when the methylation level is high. We believe our findings addresses a timely issue regarding the relation between DNA methylation and mutation in terms of gene expression in tumorigenesis.

## Additional file

Additional file 1 Supplementary file contains Supplementary Figure S1–4 and Table S1–2. (PDF 2170 kb)
