# Supplementary material for: Subtype-specific CpG island shore methylation and mutation patterns in 30 breast cancer cell lines
Source: BMC Syst Biol. 2016 Dec 23;10(Suppl 4):116. doi: 10.1186/s12918-016-0356-2 (PMC5259919; doi:10.1186/s12918-016-0356-2)
Supplement: Additional file 1 — Supplementary file contains Supplementary Figure S1–4 and Table S1–2. (PDF 2170 kb) [file 12918_2016_356_MOESM1_ESM.pdf]

Supplementary Data

Supplementary Figure S1

Supplementary Figure S2

Supplementary Figure S3

Supplementary Figure S4

Supplementary Table S1

Supplementary Table S2

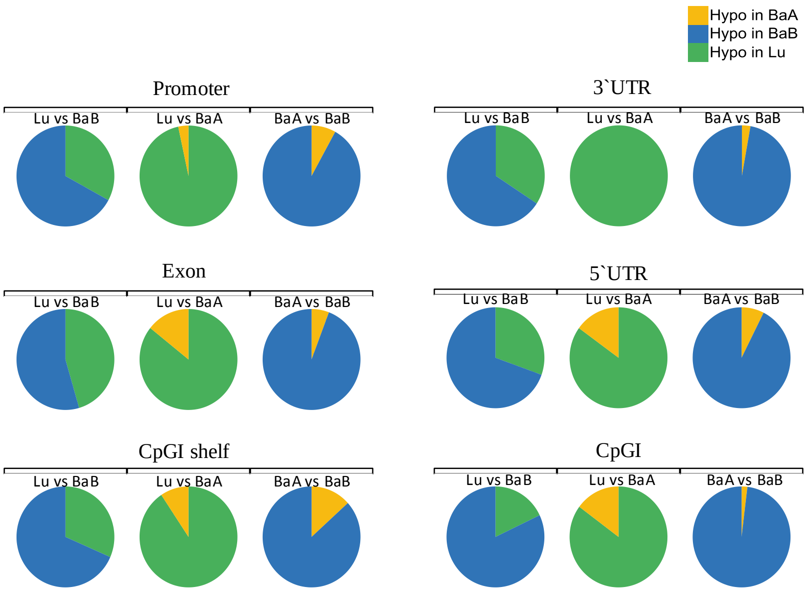

Supplementary Figure S1. Ratio of hypo methylation in various genomic regions for each subtype comparison. Each color represents hypo methylation ratio of certain tumor subtype among differentially methylated bins.

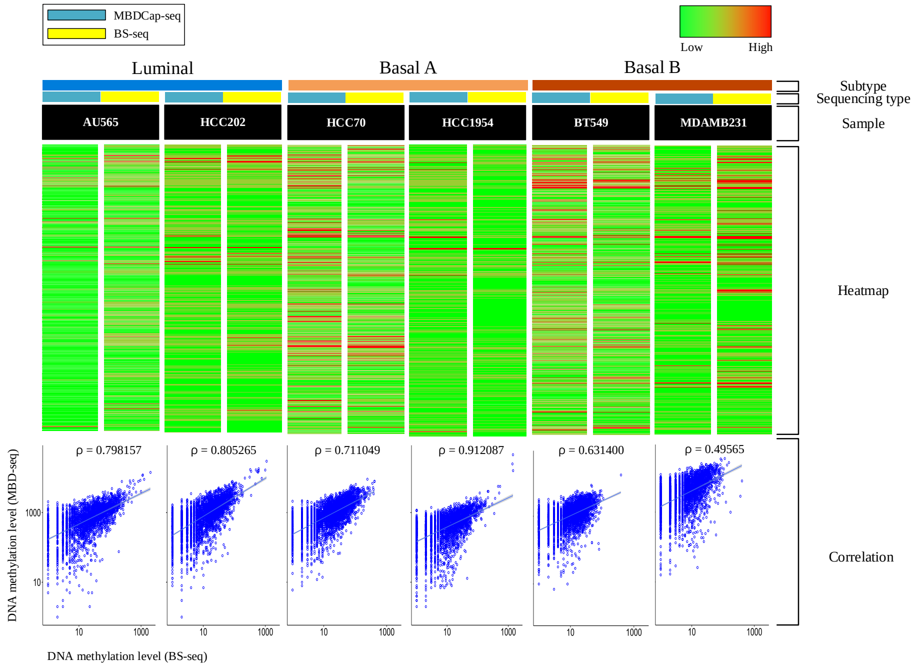

Supplementary Figure S2. Comparison of DNA methylation using MBDCap-sequencing and bisulfite sequencing. Methylation status at same genomic positions from both sequencing results are aligned side by side and illustrated as heatmaps. Dot plots represent correlations between the two approaches. X-axis and y-axis of dot plots represent log scale of methylation level measured by MBDcap-seq and BS-seq respectively with 2kb binning.

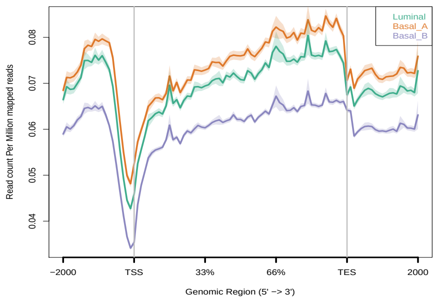

(a) Average methylation in genebody region

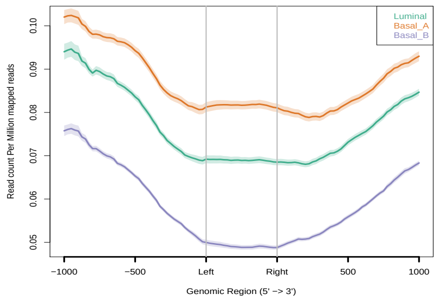

(c) Average methylation in DHS region

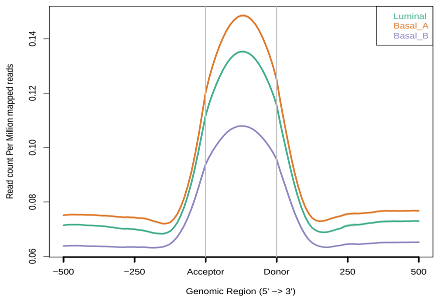

(b) Average methylation in exon region

Supplementary Figure S3. Average genome wide methylation plot of various genomic regions. X-axis represents relative range of the region and their flanking region, and y-axis show average methylation level measured by MBDcap sequencing.

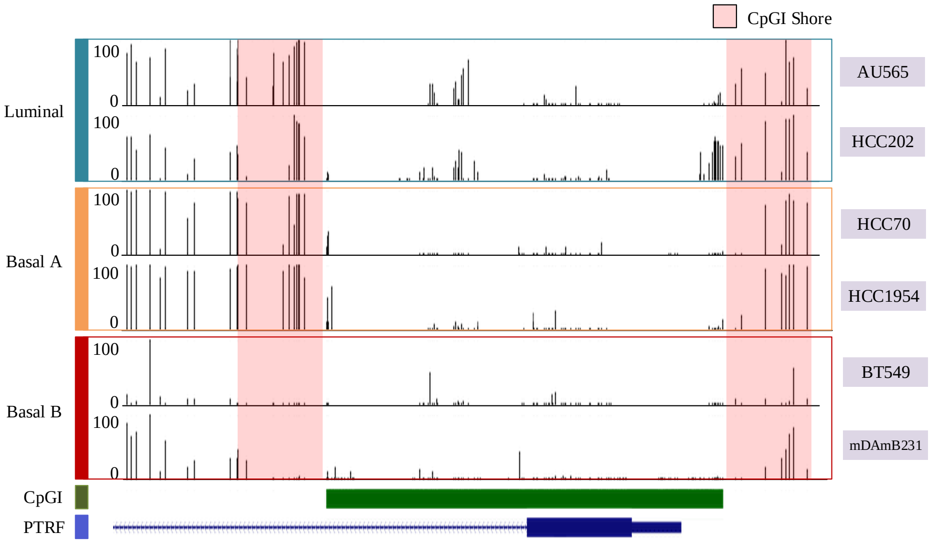

Supplementary Figure S4. Validation of methylation difference of CpG shore and promoter overlapped region using bisulfite treated sequencing. The *PTRF* gene at single base pair resolution across the tumor subtypes is illustrated. In agreement with MBDcap sequencing, hypermethylation is observed from luminal and basal A whereas hypomethylation is observed in Basal B subtype in CpGI shore region.

Supplementary Table S1. 30 breast cancer cell line with tumor subtypes. The cell lines are classified by Neve *et al. (2006).*

Cell line	Tumor Subtype	ER	HER2	PR
AU565†	Luminal	-	+	-
HCC1428	Luminal	+	.	+
HCC202†	Luminal	-	+	-
SUM185PE	Luminal	-	.	-
600MPE	Luminal	+	.	-
SKBR3	Luminal	-	+	-
MDAMB453	Luminal	-	.	-
SUM52PE	Luminal	+	.	-
MDAMB175VII	Luminal	+	.	-
HCC2185	Luminal	-	.	-
LY2	Luminal	+	.	-
MCF7	Luminal	+	.	+
BT474	Luminal	+	+	+
HCC1937	Basal A	-	.	-
HCC70†	Basal A	-	.	-
HCC1954†	Basal A	-	+	-
HCC1569	Basal A	-	+	-
SUM225CWN	Basal A	-	+	-
HCC1143	Basal A	-	.	-
BT20	Basal A	-	.	-
BT549†	Basal B	-	.	-
MDAMB231†	Basal B	-	.	-
MDAMB436	Basal B	-	.	-
HCC1500	Basal B	-	.	-
SUM1315MO2	Basal B	-	.	-
HS578T	Basal B	-	.	-
MCF12A	Basal B	-	.	-
SUM149PT	Basal B	-	.	-
MCF10A	Basal B	-	.	-
SUM159PT	Basal B	-	.	-

†Validated their methylation status by targeted bisulfite sequencing.

Supplementary Table S2. Differential methylation at 55 genes (tested by Kruskal wallis test and FDR < 0.1) at TFBS in promoter CpGI shore region and inversely correlated (Spearman's rho < -0.5) gene expressions. Heatmap represents TFBS methylation and associated gene expression status.

Low – High

Gene	TFBS methylation			Gene expression			Hypomethylated in subtype	Polycomb-associated H3K27me3 / DHS	Function / Feature
	Lu	BaA	BaB	Lu	BaA	BaB			
AKAP12							Basal B	Polycomb, DHS	Control of cell proliferation
ALDH4A1							Luminal	Polycomb, DHS	Inhibition of cell growth
CAV1							Basal B	DHS	Caveolae associated
CDS1							Luminal	DHS	Regulate phosphatidylinositol
CLDN7							Basal A	DHS	Claudin family
CRIM1							Basal B	Polycomb, DHS	Tissue development
CTNNAL1							Basal B	DHS	Rho pathway signaling
DSC2							Basal A	Polycomb	Calcium-dependent cell adhesion
DUSP1							Basal B	DHS	Cellular proliferation
ECHDC1							Basal B	DHS	Breast cancer risk
FAM114A1							Basal A	DHS	Neuronal cell development
FGFR4							Luminal	DHS	Breast fibroadenoma
FSCN1							Basal B	DHS	Fascin family of actin-binding
FTL							Basal B	DHS	Iron Storing
GDF15							Basal B	DHS	Growth differentiation factor
GPX1							Basal B	.	ALA repeats - breast cancer
GRHL2							Basal A	DHS	Epithelial phenotype - breast cancer
GSTP1							Basal B	Polycomb, DHS	Promoter methyl - clinical parameter
HES1							Basal A	DHS	Control of cell proliferation
IGFBP7							Basal B	DHS	Apoptosis
IRS1							Luminal	Polycomb, DHS	Tumor cell survival and growth
JUP							Basal A	DHS	Plakoglobin, cell dissemination
KRT19							Luminal	DHS	Cell proliferation - breast cancer
LDHB							Basal B	DHS	Essential gene - TNBC
MICA							Basal B	.	Immune escape
MLPH							Luminal	Polycomb, DHS	Basal-like classification
MT2A							Basal B	DHS	Risk of ductal breast cancer
MTUS1							Basal A	Polycomb	Breast cancer cell proliferation
MYO6							Basal A	DHS	Intracellular processes, cell migration
NEBL							Basal A	DHS	Assembly of focal adhesion
NT5E							Basal B	DHS	CpGI methyl - biomarker
PALLD							Basal B	Polycomb	Invasive motility in breast cancer
PGM1							Basal A	DHS	Phosphohexose mutase family
PHLDA1							Basal B	DHS	Formation of ER+ mammospheres
PLAUR							Basal B	DHS	Invasive ductal breast carcinoma
PLXNB1							Luminal	Polycomb, DHS	Promote breast cancer metastasis
PMAIP1							Basal A	Polycomb, DHS	Apoptosis
PTPRK							Basal A	DHS	Adhesion, invasion of breast cancer
PTRF							Basal B	DHS	Caveolae associated
PTX3							Basal B	DHS	Bone metastatic breast cancer
PVRL3							Basal B	DHS	Cell-cell adhesion
RBM47							Basal A	DHS	Metastatic traits
SLC16A1							Basal B	Polycomb, DHS	lactate transporter
SLC1A4							Basal B	Polycomb, DHS	Disorder of intellectual disability
SLC2A10							Basal B	Polycomb, DHS	Cell proliferation in luminal subtype
SPRY2							Basal B	Polycomb, DHS	Aberrant growth factor signaling
STAG2							Basal A	Polycomb, DHS	Cell division
TAF1A							Basal B	Polycomb, DHS	TATA box-binding
TCF4							Basal B	Polycomb	Chemosensitivity
TGFB1							Basal B	DHS	Growth factor
TRIL							Luminal	DHS	Lipopolysaccharide
TRPS1							Luminal	Polycomb, DHS	E-cadherin and ?-catenin status
TTC39A							Luminal	Polycomb, DHS	Non-annotated
VIM							Basal B	Polycomb, DHS	Cell adhesion and migration
XBP1							Luminal	Polycomb, DHS	Tumorigenicity and progression

55% genes are hypo methylated in basal B tumor subtype

DHS : DNase I hypersensitive site, TFBS : Transcription factor binding site
